# Genetic Association of NPY Gene Polymorphisms with Dampness-Phlegm Pattern in Korean Stroke Patients

**DOI:** 10.1155/2012/109796

**Published:** 2011-10-29

**Authors:** Mi Mi Ko, Byoung Kab Kang, Ji Hye Lim, Myeong Soo Lee, Min Ho Cha

**Affiliations:** Brain Disease Research Center, and Division of Standard Research, Korea Institute of Oriental Medicine, 1672 Yuseongdae-ro, Yuseong-gu, Daejeon 305-811, Republic of Korea

## Abstract

Neuropeptide Y (NPY), which is widely expressed in both the central and peripheral nervous systems, has an important role in a variety of biological fields. In this study, we analyzed the distribution of NPY polymorphisms in dampness-phlegm pattern and non-dampness-phlegm pattern in elderly Korean subjects with cerebral infarction (CI). A total of 1.097 subjects (498 normal subjects and 599 CI patients, including 198 with dampness-phlegm pattern and 401 with non-dampness-phlegm pattern) participated in this study. Genotyping for five SNPs (G-1484A, C-1471T, C-399T, A1201G, and C5325T) was conducted by primer extension. The results were statistically analyzed for genetic association of NPY-polymorphisms with normal versus dampness-phlegm pattern or non-dampness-phlegm pattern subjects. Among the five SNPs tested, the T allele of C-399T has a negative association with the dampness-phlegm pattern and is marked by a decrease in serum cholesterol levels. Furthermore, serum cholesterol levels were significantly higher in dampness-phlegm pattern patients than in non-dampness-phlegm pattern patients.In this study, for the first time, the association of NPY polymorphisms with pattern identification (PI) of traditional Korean medicine (TKM) was analyzed in a large CI patient population.

## 1. Introduction

Neuropeptide Y (NPY), first identified in mammalian brains in the 1980s, is a neuropeptide consisting of 36 amino acids [1, 2] that exhibits diverse biological functions [3]. Most of the NPY protein is secreted by the peripheral or central nervous systems [1] and is involved in multiple physiological processes and pathological conditions, including memory, stress, overeating and obesity, pain regulation, neural regulation, Alzheimer's disease, regulation of blood pressure, and cerebral circulation [3–13]. Direct or indirect effects of NPY have been particularly implicated in obesity and metabolic syndromes. Largely secreted NPY stimulates eating and drives overeating-induced obesity [14]. Furthermore, the expression and secretion of NPY by sympathetic nerves and fat tissue was recently shown to activate proliferation and adipogenesis of preadipocytes in fat tissue [15–17].

The NPY gene, located at chromosome 7p15.1, has twenty-five polymorphisms in its promoter region and seven within its exons (NT_007819.17). The L7P SNP has been associated with type 2 diabetes mellitus, alcoholism, and obesity [18–22]. Another functional SNP, C-399T, in which the T allele exhibits lower transcriptional activity than the C allele, was also associated with schizophrenia [23] and non-Hodgkin's lymphoma [24]. Recently, Kim et al. reported that this SNP was significantly associated with ischemic stroke in the Korean elderly population [25], with Yu et al. showing the same results in Chinese subjects [26].

Traditional Korean medicine (TKM), which is similar to traditional Chinese medicine (TCM), categorizes stroke into internal disease symptoms. Pattern identification (PI) is a diagnostic system that entails a comprehensive analysis of symptoms and signs, with implications for determining the cause, nature, and location of the illness; the patient's physical condition; the patient's treatment [27, 28]. Go previously reported that the PI of stroke in TKM can be classified into five types: fire heat pattern, dampness-phlegm pattern, blood stasis pattern, yin deficiency pattern and Qi deficiency pattern [29]. Among these, subjects with dampness-phlegm pattern tend to be obese and have hyperlipidemia [30]. PI is affected by environmental and hereditary factors. Some reports have suggested a relationship between genetic variation and PI among Chinese, Korean, and Japanese populations [31–34].

In this study, we analyzed the distribution of NPY polymorphisms in dampness-phlegm pattern and non-dampness-phlegm pattern in elderly Korean subjects.

## 2. Materials and Methods

### 2.1. Study Subjects

Patients with cerebral infarction (CI) were admitted into twelve Korean oriental medical hospitals participating in this study: Kyung Hee Oriental Medical Center (Seoul), Kyung Hee East-West Neo Medical Center (Seoul), Dong Guk International Hospital (Kyunggi-do), Kyung Won Oriental Medical Hospitals (Seoul and Incheon), Dong Seo Oriental Medical Hospital (Seoul), Dae Jeon Oriental Medical Hospital (Daejeon), Dong Sin Oriental Medical Hospital (Gwangju and Jeollanam-do), Won Kwang Oriental Medical Hospital (Jeollabuk-do), Woo Suk Oriental Medical Hospital (Jeollabuk-do), and Sang Ji Oriental Medical Hospital (Gangwon-do). CI was confirmed by magnetic resonance imaging (MRI) or magnetic resonance angiography (MRA). After obtaining informed consent from all subjects, clinical data were collected, and syndromes were differentiated classified using “stroke PI case report form.” PI diagnosis of each patient was determined by two expert TKM doctors, and subjects receiving differing opinions from two doctors were excluded [27]. Patients with histories of transient ischemic attack (TIA), diabetes, hyperlipidemia, and heart disease were excluded, as were patients with a previous stroke or a traumatic stroke. 

Normal subjects were recruited from Dae Jeon Oriental Medical Hospital and Won Kwang Oriental Medical Hospital, and stroke status was confirmed by MRI. Subjects with a history of stroke, TIA, diabetes, hyperlipidemia and heart disease were excluded. Four hundred ninety-eight normal subjects and five hundred ninety-nine CI patients classified as having dampness-phlegm pattern (*N* = 198) or non-dampness-phlegm pattern (*N* = 401) participated in this study. The general characteristics of normal subjects and CI patients are shown in Supplemental Table 1 (See in Supplementary Material available online at doi:10.1155/2012/109796). This study was approved by the Institutional Review Boards of the Korean institute of Oriental Medicine and by each of the Oriental Medical Hospitals.

### 2.2. Preparation of Genomic DNA and Identification of SNPs

Genomic DNA of each subject was extracted from whole blood using a GeneAll Genomic DNA Extraction Kit (GeneAll, Seoul, Korea). The promoter region (2 kb) and all exons of the NPY gene were sequenced in 24 Korean genomic DNA samples using an ABI PRISM 3700 DNA analyzer (Applied Biosystems, Foster City, Calif, USA). Primer sets used in the amplification and sequencing analyses were designed on the basis of the reference genome sequence for NPY (NC_000007). Information concerning the primers for the amplification and sequencing of the NPY gene is shown in Supplemental Table 2.

Genotyping of SNPs in all subjects was conducted using the SNaPshot multiplex system (Applied Biosystem, FosterCity, CA, USA) according to the manufacturer's protocol. Primer and NPY SNP genotyping probe sequences are shown in Supplemental Table 3. 

Hardy-Weinberg equilibrium tests were employed to determine whether individual SNPs were in equilibrium at each locus, and linkage disequilibrium (LD) coefficients, |*D*′| and *r* ^2^, were evaluated to measure LD between all pairs of loci [35]. The frequencies of SNPs and LD coefficients were inferred using the HapAnalyzer program v1.0.

### 2.3. Statistical Analysis

Data were statistically analyzed with SAS software, version 9.1.3 (SAS Institute Inc., Cary, NC). All continuous variables were subjected to a Kolmogorov-Smirnov normality test. Differences in continuous variables were determined by parametric (Student's *t*-test) or nonparametric (Wilcoxon rank sum test) test. Categorical variables were compared with a chi-square test or Fisher's exact test.

Multiple logistic regression adjusted for age, sex, smoking status, and drinking status was performed to estimate the association of SNPs with normal versus dampness-phlegm pattern (or non-dampness-phlegm pattern), as well as odds ratios (ORs) with 95% confidence intervals (95% CI). To investigate whether the C-399T polymorphism is associated with the clinical parameters of the normal subjects, we performed a statistical analysis using a general linear model adjusted for age, sex, smoking status, and drinking status. Statistical significance was set at *P* < 0.05.

## 3. Results

General characteristics of normal and CI patients are shown in Supplemental Table 1 and are similar to our previous study [25]. The clinical differences between the CI patients classified as dampness-phlegm pattern or non-dampness-phlegm pattern according to the PI of TKM are shown in Table 1. The mean waist circumference of the dampness-phlegm pattern patients was significantly higher than that of the normal subjects (*P* < 0.001). A higher mean waist circumference was also observed in the dampness-phlegm pattern group compared with the non-dampness-phlegm pattern group. Additionally, serum lipids, total cholesterol and LDL-cholesterol levels were higher in the dampness-phlegm pattern group than in the non-dampness-phlegm pattern group. 

**Figure 1:**
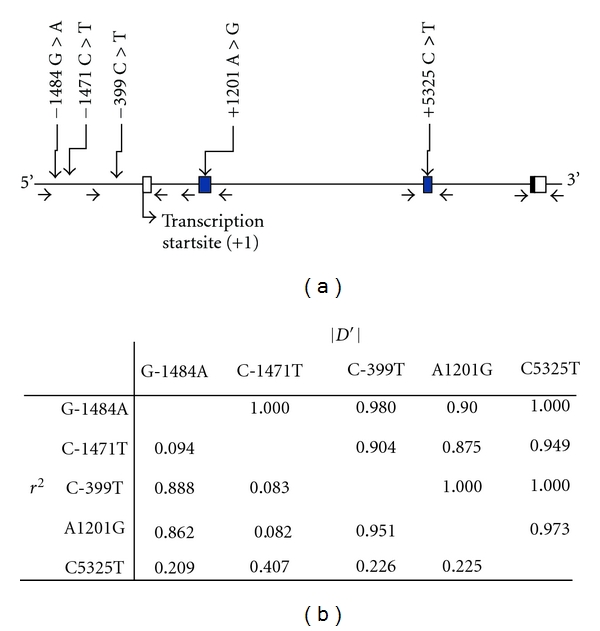
Polymorphism in NPY. (a) Location of SNPs. Open box and closed box represent non translated region and translated region, respectively. Primers for genomic amplification and sequencing are shown below the gene map. (b) Linkage coefficiency among five SNPs of NPY.

The location of five SNPs within the NPY gene are shown in Figure 1(a), and their characteristics is listed in Table 2. Three of these SNPs are in the NPY promoter, one is in exon 2, and the other is in exon 3. None of the alleles result in an amino acid change. All of the alleles were in Hardy-Weinberg equilibrium (*P* > 0.01) according to the recommended International HapMap Project guidelines. The LD coefficients between the five SNPs are shown in Figure 1(b). Among these, G-1484A, C-399T, and A1201G were slightly linked (|*D*′ | = 1 and *r* ^2^ = 0.862–0.951).

Table 3 shows the SNP distribution in the non-dampness-phlegm pattern and the dampness-phlegm pattern groups compared with the normal group. The frequency of T allele of C-399T is 27.27% in dampness-phlegm pattern, which is significantly lower than in normal (33%) and non-dampness-phlegm pattern patients (45.96%), as adjusted for sex, age, smoking, drinking and hypertension [*P* = 0.0378, OR = 0.737 (0.552–0.983)]. The ratio of subjects with T allele in the dampness-phlegm pattern group (45.96%) is also smaller than in the normal (53.25%) and non-dampness-phlegm pattern groups (56.0%) [*P* = 0.0315, OR = 0.663 (0.456–0.964)]. In contrast, subjects with T allele of C5325T at exon 3 exhibited significantly more dampness-phlegm pattern than normal and non-dampness-phlegm pattern in the dominant model.

Table 4 shows the comparison of obesity phenotypes and serum lipids in C-399T genotypes. The level of total cholesterol and LDL-cholesterol in normal subjects with TT type at C-399T was significantly lower than subjects with C allele (*P* = 0.0111 and *P* = 0.0186, resp.). Conversely, triglycerides were slightly increased in the dominant model. 

## 4. Discussion

PI is the basic system for diagnosis of patients in TKM, and it entails a systematic analysis of the patient's physical condition with implications for determination of the cause, nature, location, and treatment of the illness. The PI can lead TKM doctors to provide their patients with individualized treatment, thereby increasing the effectiveness of TKM and minimizing its adverse effects. 

In Korea, several questionnaires for the standardization of PI have been developed, the recent establishment of five standard PI subtypes for stroke: fire heat pattern, dampness-phlegm pattern, blood stasis pattern, Yin deficiency pattern and Qi deficiency pattern [29, 36]. Among these, the dampness-phlegm pattern is characterized by its impediment to Qi movement and its turbidity, heaviness, stickiness, and downward-flowing properties [17]. Moreover, Kim et al. [27] reported that pale tongue, slippery pulse, and overweight status are major factors positively associated with dampness-phlegm pattern. Other studies showed that Korean stroke patients with dampness-phlegm pattern had increased serum cholesterol levels [30]. We also obtained similar results in this study (Table 1). Collectively, these factors suggest that genes or genetic variation related with obesity might be associated with dampness-phlegm pattern in stroke. 

In the current study, we examined the association of the dampness-phlegm pattern of stroke with five SNPs of the NPY gene among Korean CI patients and found that the C-399T SNP was negatively associated with dampness-phlegm pattern (OR = 0.663) (Table 3). 

NPY, which is widely expressed in both the central and peripheral nervous systems, has an important role in the hypothalamic regulation of energy balance by stimulating food intake and favoring energy storage through increased lipoprotein lipase activity in white adipose tissue [14]. 

Many groups have studied the association of NPY polymorphisms with obesity and/or serum lipid levels. Karvonen et al. [37] reported that the presence of the P allele of L7P SNP was associated with higher serum levels of total and LDL-cholesterol in obese and normal-weight subjects, and others have shown that L7P SNP is associated with increased body mass index (BMI) [38]. However, this polymorphism has not been found in Asian populations [39, 40]. 

Plasma NPY level is positively correlated with total cholesterol and LDL-cholesterol levels [41], and our results show that the C-399T SNP is significantly associated with decreased serum total cholesterol and LDL-cholesterol in normal subjects (Table 4). Recently, four studies found that the C-399T SNP affects NPY level. We previously reported that the T allele of C-399T decreases NPY transcriptional activity and that the plasma NPY level of subjects with the TT type was significantly decreased compared with subjects with the CC or CT type [25]. In addition, three other groups documented the allele-specific effects of the C-399T polymorphism on NPY gene expression [42, 43]. Taken together, these data suggest that subjects with the TT type of C-399T may have lower levels of serum total cholesterol and LDL-cholesterol, due to decreased plasma NPY levels. 

In this study, for the first time, the association of NPY polymorphisms with PI of TKM was analyzed in a large CI patient population. Among the five SNPs, the T allele of C-399T has a negative association with dampness-phlegm pattern by decreased levels of serum cholesterol, which were significantly higher in dampness-phlegm pattern patients compared with non-dampness-phlegm pattern patients. It is clear that the findings of this study should be interpreted within the context of its limitations. Our findings are not appropriate for extrapolation to the general population. The observed associations, therefore, will require further confirmation in other subject groups.

## Supplementary Material

Supplementary Table 1: This summary is the general characteristics of normal subjects and CI patients. The results are shown that waist circumference and waist hip ratio were significantly higher in patients than normal subjects. In serum, fasting blood sugar also increased in patients.
Supplementary Table 2: This information is concerning the primers for the amplification and sequencing of the NPY gene.
Supplementary Table 3: Primer and NPY SNP genotyping probe sequences.

## Figures and Tables

**Table 1:** Demographic parameters of study subjects.

Characteristics	Normal	Non-dampness-phlegm	sig.^a^	Dampness-phlegm	sig.^b^
*N*	498	401		198	
*Anthropometric characteristics*					
Sex (M/F)	215/283	226/175	< **.0001**	104/94	< **.05**
Age (year)	60.76 ± 9.77	67.02 ± 11.94	< **.0001**	64.7±12.81	< **.0001**
Smoking (none/stop/active)	241/214/43	216/81/104	< **.0001**	117/21/60	< **.0001**
Drinking (none/stop/active)	298/31/168	199/59/143	< **.0001**	114/14/69	NS
WHR	0.88 ± 0.06	0.93 ± 0.07	< **.0001**	0.93 ± 0.05	< **.0001**
Waist circumference (cm)	83.61 ± 8.38	84.61 ± 9.79	NS	87.13 ± 9.82	< **.001**

*TOAST classification*					
LAA	—	64	ND	30	ND
CE	—	15		8	
SVO	—	237		123	
SOE	—	9		2	
SUE	—	14		4	

*Medical history*					
Hypertension (Yes, %)	60 (12.07)	217 (54.94)	< **.0001**	120 (60.61)	< **.0001**

*Blood parameter*					
GOP (U/mL)	27.56 ± 19.97	27.31 ± 22.5	NS	26.08 ± 16.07	NS
GPT (U/mL)	26.04 ± 20.26	25.6 ± 24.29	NS	26.19 ± 23.37	NS
Total cholesterol (mg/dL)	202.23 ± 40.04	181.5 ± 43.68	< **.0001**	192.83 ± 43.95	< **.01**
Triglyceride (mg/dL)	139.88 ± 69.80	149.86 ± 95.72	NS	150.75 ± 101.28	NS
HDL-cholesterol (mg/dL)	52.28 ± 12.69	46.1 ± 14.33	< **.0001**	44.57 ± 12.44	< **.0001**
LDL-cholesterol (mg/dL)	121.19 ± 36.70	109.42 ± 36.75	< **.0001**	121.21 ± 40.35	NS
Bloog sugar (mg/dL)	99.75 ± 10.79	112.05 ± 34.48	< **.0001**	109.81 ± 29.53	< **.0001**

All results except sex, TOAST categories, smoking, drinking, and hypertension are expressed as mean ± SD for continuous variables. NS: not significant. ND: not done. WHR: waist hip ratio. TOAST: trial of ORG 10172 in acute stroke treatment. LAA: large-artery atherosclerosis. CE: cardioembolism. SVO: small-vessel occlusion. SOE: stroke of other etiology. SUE: stroke of undetermined etiology. GOP: glutamate oxaloacetate transaminase. GPT: glutamate pyruvate transaminase. sig.^a^: *P* value of normal versus non-dampness-phlegm using a Student's *t*-test or Wilcoxon rank-sum test in continuous variables, chi-square test, or Fisher's exact test in categorical variables. sig.^b^: *P* value of normal versus dampness-phlegm using a Student's *t*-test or Wilcoxon rank sum test in continuous variables, chi-square test, or Fisher's exact test in categorical variables. *P* values with statistical significance were presented in bold.

**Table 2:** Characteristics of SNPs identified by genomic sequencing of the promoter, exon of NPY in normal group.

SNP	rs no.	Position	Location relative to transcription start site	Nucleotide change	Location relative to *p* terminus of chromosome	Minor allele frequency	HWE *p *
G-1484A	rs16149	Promoter	−2535	G>A	24,322,325	0.314	0.2624
C-1471T	rs16148	Promoter	−2522	C>T	24,322,338	0.171	0.2733
C-399T	rs16147	Promoter	−1450	C>T	24,323,410	0.331	0.0709
A1201G	rs5573	Exon 2	150	A>G	24,325,009	0.342	0.0417
C5325T	rs5574	Exon 3	4274	C>T	24,329,133	0.314	0.1836

**Table 3:** Genotype distribution of NPY polymorphism of dampness-phlegm and non-dampness-phlegm.

Model	SNPs	Genotype	Normal	Non-dampness-phlegm	^c^OR [95% CI]	sig.^a^	Dampness-phlegm	^d^OR [95% CI]	sig.^b^
Allele	G-1484A	G	682 (68.75)	544 (68)	0.988	0.9145	294 (74.24)	0.748	0.0524
		A	310 (31.25)	256 (32)	[0.787, 1.240]		102 (25.76)	[0.558, 1.003]	
	C-1471T	C	824 (82.90)	663 (83.29)	1.051	0.7314	323 (81.98)	0.931	0.6867
		T	170 (17.10)	133 (16.71)	[0.791, 1.398]		71 (18.02)	[0.656, 1.320]	
	C-399T	C	666 (67.0)	535 (66.88)	1.007	0.9511	288 (72.73)	0.737	**0.0378**
		T	328 (33.0)	265 (33.13)	[0.804, 1.261]		108 (27.27)	[0.552, 0.983]	
	A1201G	A	653 (65.83)	497 (62.13)	0.868	0.2099	272 (68.69)	0.879	0.3666
		G	339 (34.17)	303 (37.88)	[0.696, 1.083]		124 (31.31)	[0.664, 1.163]	
	C5325T	C	682 (68.75)	534 (66.75)	0.851	0.1658	250 (63.13)	1.250	0.1131
		T	310 (31.25)	266 (33.25)	[0.678, 1.069]		146 (36.87)	[0.948, 1.648]	

^†^Do	G-1484A	GG	240 (48.39)	181 (45.25)	0.93	0.6363	110 (55.56)	0.697	0.0583
		GA+AA	256 (51.61)	219 (54.75)	[0.688, 1.257]		88 (44.44)	[0.480, 1.013]	
	C-1471T	CC	338 (68.01)	276 (69.35)	1.058	0.7319	135 (68.53)	0.918	0.6762
		CT+TT	159 (31.99)	122 (30.65)	[0.765, 1.465]		62 (31.47)	[0.614, 1.373]	
	C-399T	CC	232 (46.68)	176 (44)	0.953	0.7569	107 (54.04)	0.663	**0.0315**
		CT+TT	265 (53.32)	224 (56)	[0.705, 1.29]		91 (45.96)	[0.456, 0.964]	
	A1201G	AA	225 (45.36)	158 (39.5)	0.821	0.2059	95 (47.98)	0.862	0.4357
		AG+GG	271 (54.64)	242(60.5)	[0.605, 1.114]		103 (52.02)	[0.594, 1.252]	
	C5325T	CC	241 (48.59)	179 (44.75)	0.794	0.1335	76 (38.38)	1.557	**0.0210**
		CT+TT	255 (51.41)	221 (55.25)	[0.587, 1.073]		122 (61.62)	[1.069, 2.269]	

^†^R	G-1484A	GG+GA	442 (89.11)	363 (90.75)	1.148	0.5868	184 (92.93)	0.700	0.2961
		AA	54(10.89)	37 (9.25)	[0.697, 1.89]		14 (7.07)	[0.359, 1.366]	
	C-1471T	CC+CT	486 (97.79)	387 (97.24)	1.077	0.8826	188 (95.43)	0.937	0.9019
		TT	11 (2.21)	11 (2.76)	[0.404, 2.868]		9 (4.57)	[0.330, 2.656]	
	C-399T	CC+CT	434 (87.32)	359 (89.75)	1.157	0.5444	181 (91.41)	0.750	0.3579
		TT	63 (12.68)	41 (10.25)	[0.722, 1.853]		17 (8.59)	[0.406, 1.385]	
	A1201G	AA+AG	428 (86.29)	339 (84.75)	0.869	0.518	177 (89.39)	0.830	0.5201
		GG	68 (13.71)	61 (15.25)	[0.568, 1.33]		21 (10.61)	[0.469, 1.466]	
	C5325T	CC+CT	441 (88.91)	355 (88.75)	0.878	0.5966	174 (87.88)	0.907	0.7457
		TT	55 (11.09)	45 (11.25)	[0.544, 1.419]		24(12.12)	[0.503, 1.636]	

Data are presented as frequencies (percentages). ^c^ORs after adjustment for sex, age, drinking, and smoking with normal versus non-dampness-phlegm. ^d^ORs after adjustment for sex, age, drinking, and smoking with normal versus dampness-phlegm. sig.^a^: *P* value of normal versus non-dampness-phlegm using multiple logistic regression analysis. sig.^b^: *P* value of normal versus dampness-phlegm using multiple logistic regression analysis. ^†^Do and R denote dominant and recessive models, respectively. CI: confidence interval. *P*-values with statistical significance were presented in bold (<0.05).

**Table 4:** Association of NPY polymorphism (C-399T) with clinical parameters of normal subjects.

Variable	Genotype			*P*		
	CC (*n* = 232)	CT (*n* = 202)	TT (*n* = 63)	^†^Co	^†^Do	^†^R
Waist	82.8 ± 8.43	84.46 ± 8.5	83.58 ± 7.47	0.4988	0.3007	0.8857
WHR	0.88 ± 0.07	0.88 ± 0.05	0.87 ± 0.05	0.4535	0.3418	0.2656
Total cholesterol	199.27 ± 37.97	209.11 ± 42.71	190.79 ± 35.25	**0.0019**	0.1748	**0.0111**
Triglyceride	132.15 ± 66.49	147.14 ± 74.71	143.49 ± 63.13	0.0869	**0.03**	0.697
HDL-cholesterol	52.9 ± 13.05	52.79 ± 12.55	50.89 ± 11.86	0.3289	0.3076	0.1666
LDL-cholesterol	119.32 ± 35.21	126.38 ± 39.09	111.2 ± 31.91	**0.0076**	0.2913	**0.0186**
Blood sugar	99.33 ± 9.77	99.95 ± 11.63	100.7 ± 11.76	0.4249	0.1984	0.4885

Data are presented as mean ± S.D. *P* values adjusted odds ratio, adjustment for sex, age, drinking, and smoking using a general linear model. Statistical significance was presented in bold (<0.05). ^†^Co, Do, and R denote codominant, dominant, and recessive models, respectively.
