# Supplementary material for: Genetic Association of NPY Gene Polymorphisms with Dampness-Phlegm Pattern in Korean Stroke Patients
Source: Evid Based Complement Alternat Med. 2011 Oct 29;2012:109796. doi: 10.1155/2012/109796 (PMC3205675; doi:10.1155/2012/109796)
Supplement: Supplementary file 1 — Supplementary Table 1: This summary is the general characteristics of normal subjects and CI patients. The results are shown that waist circumference and waist hip ratio were significantly higher in patients than normal subjects. In serum, fasting blood sugar also increased in patients. Supplementary Table 2: This information is concerning the primers for the amplification and sequencing of the NPY gene. Supplementary Table 3: Primer and NPY SNP genotyping probe sequences. [file 109796.f1.pdf]

Supplemental table 1. Demographic parameters of study subjects

Characteristics	Normal	Case*	*sig.*
N	498	599	
anthropometric characteristics			
sex (M/F)	215/283	330/269	**<.0001**
age (year)	60.76±9.77	66.25±12.28	**<.0001**
smoking (none/stop/active)	241/214/43	333/102/164	**<.0001**
drinking (none/stop/active)	298/31/168	313/73/212	**0.0013**
WHR	0.88±0.06	0.93±0.06	**<.0001**
weight (kg)	61.74±9.15	61.43±11.34	NS
waist circumference (cm)	83.61±8.38	85.56±9.86	**0.0026**
TOAST classification			
LAA	-	94	ND
CE	-	23	
SVO	-	360	
SOE	-	11	
SUE	-	18	
medical history			
hypertension (Yes,%)	60(12.07)	337(56.83)	**<.0001**
blood parameter			
GOP (U/ml)	27.56±19.97	26.91±20.59	NS
GPT (U/ml)	26.04±20.26	25.79±23.97	NS
total cholesterol (mg/dL)	202.23±40.04	185.32±44.06	**<.0001**
triglyceride (mg/dL)	139.88±69.80	150.17±97.59	NS
HDL-cholesterol (mg/dL)	52.28±12.69	45.55±13.68	**<.0001**
LDL-cholesterol (mg/dL)	121.19±36.70	113.69±38.48	**0.0019**
bloog sugar (mg/dL)	99.75±10.79	111.18±32.64	**<.0001**

All results except sex, TOAST categories, smoking, drinking and hypertension are expressed as mean ± SD for continuous variables. NS: not significant. ND: not done. WHR: waist hip ratio. TOAST: Trial of ORG 10172 in Acute Stroke Treatment. LAA: large-artery atherosclerosis. CE: cardioembolism. SVO: small-vessel occlusion. SOE: stroke of other etiology. SUE: stroke of undetermined etiology. GOP: glutamate oxaloacetate transaminase. GPT: glutamate pyruvate transaminase. * : cerebral infarction patients. *sig.* : P-value using a Student's t-test or Wilcoxon rank-sum test in continuous variables, chi-square test or Fisher's exact test in categorical variables. P-values with statistical significance were presented in bold.

Supplemental table 2. Primer sets for PCR amplification and sequencing in promoter and exon regions of *NPY* gene

region	PCR amplification primer			sequencing primer		
	location	direction	sequence	location	direction	sequencing
promoter + exon1	,-2071	forward	CACATGGACCTTTACCGGTT	-2071	forward	CACATGGACCTTTACCGGTT
	1349	reverse	GTGCCTGATACTGTCCTGC	-1749	forward	CCTCAGAATAAGTTCTTATTC
				-1239	forward	AGAATCACTTGTCCTTTTAGCA
				-280	reverse	CCAAAGCCCAAGTATCTGCCCT
				+243	reverse	TAGCCGCCAAGAGGGCTGGT
Exon 2	,-2071	forward	CACATGGACCTTTACCGGTT	+1349	reverse	GTGCCTGATACTGTCCTGC
	1349	reverse	GTGCCTGATACTGTCCTGC			
Exon 3	5223	forward	CATATCCCAAATAGGAGACTAT	+5223	forward	CATATCCCAAATAGGAGACTAT
	5482	reverse	CCCTAGTCTAGAAGCCTGGT			
Exon 4	7730	forward	CAGTTCCCGGTCATCTTTCA	+7760	reverse	CTGAATCTGCTAGTTGAGGG
	,+7760	reverse	CTGAATCTGCTAGTTGAGGG			

Supplemental table 3. Primer sets for PCR amplification and genotyping of each SNPs

SNPs		PCR amplification primer	genotyping primer
G-1484A	forward	GAATCAAGCTATGAACCTGCTATC	aatagaagatgttttgcacttttacc
	reverse	GTGTAGAGTGTGCCCTGTACAAA	
C-1471T	forward	GAATCAAGCTATGAACCTGCTATC	agt tgctcgggggtgcttac
	reverse	GTGTAGAGTGTGCCCTGTACAAA	
C-399T	forward	GAATCAAGCTATGAACCTGCTATC	tcctgccaacaggactacca
	reverse	GTGTAGAGTGTGCCCTGTACAAA	
A1201G	forward	GAATCAAGCTATGAACCTGCTATC	ggacatggccagatactactc
	reverse	GTGTAGAGTGTGCCCTGTACAAA	
C5325T	forward	CATTACCCAAATAGGAGACTAT	tgaaatcagtgtctctgggct
	reverse	CACCTTGAGCTCTGCAACAATG	
